# Complete Genome Sequence of Sphingobium xenophagum PH3-15, Isolated from La Roche-Posay Thermal Water Sources

**DOI:** 10.1128/MRA.00700-21

**Published:** 2021-08-19

**Authors:** Pascal Hilaire, Sandy Contreras, Hélène Blanquart-Goudezeune, Jonathan Verbeke, Baudoin Delépine, Lucas Marmiesse, Rémi Peyraud, Stanislas C. Morand

**Affiliations:** a L’Oréal R&I, Tours, France; b GenoScreen, Lille, France; c iMean, Toulouse, France; d L’Oréal R&I, Aulnay-Sous-Bois, France; University of Maryland School of Medicine

## Abstract

We report here the complete genome sequence of Sphingobium xenophagum strain PH3-15, which was isolated from La Roche-Posay thermal water sources. The assembled 4.6-Mbp genome consisted of two chromosomes and three plasmids. These data will provide valuable information and important insights into the physiology and metabolism of this *Sphingobium* organism.

## ANNOUNCEMENT

Members of the *Sphingobium* genus are nonpathogenic, aerobic, Gram-negative, lipopolysaccharide-free alphaproteobacteria known to utilize or degrade recalcitrant natural and anthropogenic compounds (1, 2). From the systematics perspective, the genus *Sphingomonas* was renamed *Sphingobium* in 2001 (3), and the species Sphingomonas xenophaga (also known by its heterotypic synonym Sphingomonas hydrophobicum) was reclassified as Sphingobium xenophagum (4, 5). *S. xenophagum* strain PH3-15 was isolated from La Roche-Posay spa water (46°46′46″N, 0°48′27″E) in October 2006; we present here its complete genome sequence.

The strain was isolated on a tryptic soy agar plate and cultured with tryptic soy broth (TSB) medium at 30°C. The initial *Sphingobium* taxonomic identification was deciphered using a BLASTn search on the nonredundant database v2.2.29+ (6) after 16S PCR followed by Sanger sequencing. High-molecular-weight genomic DNA was extracted from an overnight culture grown in TSB at 30°C using the Gentra Puregene kit (Qiagen). Sequencing was first performed on a MinION device using an R9.4.1 flow cell (Oxford Nanopore Technologies; rapid sequencing SQK-RAD004 library with 400 ng DNA and 50-s tagmentation; base calling using Guppy v4.2.2 in 450bps_fast configuration). The long reads (2.16 Gb; 225,196 reads; *N*_50_, 19,173 bp; quality score, 9.6) were quality controlled using MinIONQC v1.4.1 (7) and filtered and trimmed using NanoFilt v2.6.0 (-q 7 -l 1000); adaptors were removed using Porechop v0.2.4 (8, 9). Then, sequencing on a HiSeq 4000 system (Illumina; Nextera XT library) generated 150-bp paired-end short reads, which were assessed using FastQC v0.11.5 (10) and cleaned using Cutadapt v1.18 (11) and Prinseq v0.20.4 (12) (parameters: -trim_qual_right 30 -trim_qual_type min -trim_qual_rule lt -trim_qual_window 7 -ns_max_n 0 -noniupac -min_qual_mean 30 -trim_left 15 -min_len 60), resulting in 2 × 12,235,539 reads (3,117 Mb). Both short and long cleaned reads were *de novo* assembled using the SPAdes v3.10.1 (13) and MaSuRCA v3.3.0 (PE = pe 600 50) (14) tools. The consensus assembly was manually curated and carefully verified using Bowtie2 v2.1.0 (15), Minimap2 v2.17 (16), and Geneious Assembler (Biomatters) by calculating the reads mapped back to contigs (RMBC) index (98.6 to 99.2% rate for the three tools). After assembly polishing using Pilon v1.23 (17), the chromosomal and plasmid coverage and circularity were validated using Genious Prime v2020.2.5 software by visualizing the reads that overlapped both the 5′ and 3′ replicon extremities. The whole-genome median coverage depth was 1,272-fold. The assembled ungapped genome is 4,577,807 bp long (GC content, 63.0%), comprising two chromosomes (3,472,664 and 623,015 bp) and three plasmids named pSH1 (263,324 bp), pSH2 (162,057 bp), and pSH3 (56,747 bp). Structural and functional annotations, carried out using the NCBI Prokaryotic Genome Annotation Pipeline v5.2 (18), identified 3 rRNA operons (5S, 16S, and 23S), 54 tRNAs, and 4,178 protein-coding sequences. Comparison with all other available *Sphingobium* genomes using FastANI v1.31 (19) showed that the closest strain is Sphingobium hydrophobicum C1 (average nucleotide identity [ANI], 0.994). Searches for mobile elements using ISsaga v2.0 (20) revealed the presence of 36 *istA* to *istB* operons belonging to the transposase-cointegrase IS*21* family (21). Finally, genes related to exopolysaccharide polymerization and export as well as to nostoxanthin pigment biosynthesis were also recovered (22–24).

### Data availability.

The complete genome sequence is available in GenBank under the accession numbers CP076556.1 to CP076560.1, BioProject accession number PRJNA600023, and BioSample accession number SAMN13781057. The version described in this paper is the first version. The raw reads have been deposited at the Sequence Read Archive under the accession number SRS8817059.
